# Induction of multiple matrix metalloproteinases in human dermal and synovial fibroblasts by *Staphylococcus aureus*: implications in the pathogenesis of septic arthritis and other soft tissue infections

**DOI:** 10.1186/ar2086

**Published:** 2006-11-27

**Authors:** Siva Kanangat, Arnold Postlethwaite, Karen Hasty, Andrew Kang, Mark Smeltzer, Whitney Appling, Dennis Schaberg

**Affiliations:** 1 Department of Medicine, University of Tennessee Health Science Center, 956 Court Avenue, Memphis, TN 38163, USA; 2 Veterans Affairs Medical Center, 1030 Jefferson Avenue, Research 151, Memphis, TN 38104, USA; 3 Department Orthopedic Surgery, University of Tennessee Health Science Center, 956 Court Avenue, Memphis, TN 38163, USA; 4 Department of Microbiology & Immunology, University of Arkansas Medical School, 4301 W. Markham Street #511, Little Rock, AR 72205, USA; 5 Greater Los Angeles Healthcare (111), 11301, Wilshire Boulevard, Los Angeles, CA 90073, USA

## Abstract

Infections of body tissue by *Staphylococcus aureus *are quickly followed by degradation of connective tissue. Patients with rheumatoid arthritis are more prone to *S. aureus*-mediated septic arthritis. Various types of collagen form the major structural matrix of different connective tissues of the body. These different collagens are degraded by specific matrix metalloproteinases (MMPs) produced by fibroblasts, other connective tissue cells, and inflammatory cells that are induced by interleukin-1 (IL-1) and tumor necrosis factor (TNF). To determine the host's contribution in the joint destruction of *S. aureus*-mediated septic arthritis, we analyzed the MMP expression profile in human dermal and synovial fibroblasts upon exposure to culture supernatant and whole cell lysates of *S. aureus*. Human dermal and synovial fibroblasts treated with cell lysate and filtered culture supernatants had significantly enhanced expression of MMP-1, MMP-2, MMP-3, MMP-7, MMP-10, and MMP-11 compared with the untreated controls (*p *< 0.05). In the *S. aureus *culture supernatant, the MMP induction activity was identified to be within the molecular-weight range of 30 to >50 kDa. The MMP expression profile was similar in fibroblasts exposed to a combination of IL-1/TNF. mRNA levels of several genes of the mitogen-activated protein kinase (MAPK) signal transduction pathway were significantly elevated in fibroblasts treated with *S. aureus *cell lysate and culture supernatant. Also, tyrosine phosphorylation was significantly higher in fibroblasts treated with *S. aureus *components. Tyrosine phosphorylation and MAPK gene expression patterns were similar in fibroblasts treated with a combination of IL-1/TNF and *S. aureus*. Mutants lacking staphylococcal accessory regulator (*Sar*) and accessory gene regulator (*Agr*), which cause significantly less severe septic arthritis in murine models, were able to induce expression of several MMP mRNA comparable with that of their isogenic parent strain but induced notably higher levels of tissue inhibitors of metalloproteinases (TIMPs). To our knowledge, this is the first report of induction of multiple MMP/TIMP expression from human dermal and synovial fibroblasts upon *S. aureus *treatment. We propose that host-derived MMPs contribute to the progressive joint destruction observed in *S. aureus*-mediated septic arthritis.

## Introduction

*Staphylococcus aureus *is the most common cause of septic arthritis (SA) [1,2]. SA has shown no change in incidence in spite of advances in antimicrobial therapy and is responsible for residual functional impairment and for a high mortality rate among debilitated patients. Risk factors include older age, diabetes mellitus, rheumatoid arthritis (RA), immunodeficiency, and a pre-existing joint disease. In SA, *S. aureus *contributes to more than two thirds of identified organisms [3,4]. In an epidemiological study of SA in an adult population of 116 patients by Abid and colleagues [5] between 1999 and 2004, *S. aureus *was the most common organism isolated from blood as well as synovial fluid (18.8%). Cleeman and colleagues [6] studied 23 culture-positive cases of SA of the glenohumeral joint between 1986 and 2000, and 52% had a different primary site of infection identified, 70% of which were *S. aureus*-positive and 17% of which were methicillin-resistant. In a retrospective analysis by Moumile and colleagues [7] of the bacterial etiology of acute osteoarticular infections in 406 children with clinically suspected osteoarticular infections, 74 (18%) had a positive bacterial culture: 38 cases of SA and 36 cases of bone infections (osteitis and osteomyelitis), the most commonly recovered pathogen being *S. aureus *(44%). Goergens and colleagues [8] reviewed the clinical presentation, management, and organisms responsible for acute hematogenous osteomyelitis (AHO) and SA in Australia between 1998 to 2002, and *S. aureus *was the most common identifiable causative organism, accounting for 76% of isolated organisms in AHO and 39% of isolated organisms in SA. *S. aureus *remains the most common organism causing AHO and SA, and multidrug-resistant *S. aureus *(MRSA) is on the increase as well. Progressive joint destruction despite appropriate antibiotic therapy and synovial fluid aspiration may indicate a potential role for host-derived proteases. Several matrix metalloproteinases (MMPs) are induced in host cells in response to infectious stimuli. Normally, MMPs assist in clearing infections, initiating immune responses, and in tissue remodeling [9]. Excessive MMPs cause matrix degradation and joint destruction as in various forms of arthritis [10].

Cytokines interleukin (IL)-1, IL-6, tumor necrosis factor (TNF)-α, and interferons (IFN-α and IFN-γ) are released from host cells in response to *S. aureus *infection and these are potent inducers of MMPs [11-15]. Staphylococcal capsule polysaccharides, toxins, cell wall-attached adhesions, and possibly also the chromosomal DNA are virulence determinants in *S. aureus *arthritis. These bacterial components might affect the innate immune response and inflammation [1]. Alternatively, the bacterial products, secreted or intracellular, could directly affect the transcriptional machinery or signal transduction pathways related to MMP expression. Previous studies have shown the induction of proteolytic enzymes in chondrocytes in response to bacteria-free culture supernatants from *S. aureus *[16]. Also, peptidoglycan (PGN) from *S. aureus *has been shown to be capable of inducing arthritis [17]. A recent study showed that *S. aureus *PGN induces MMP-1, -3, and -13 in human synovial fibroblasts [18]. Purified PGN is chemically modified and may not really represent the native PGN. Also, there is a wide variety of bacterial components, including the superantigens, cell wall components, and extracellular toxins, which could stimulate the host cells. The full potential of synovial fibroblasts in terms of multiple MMP expression in response to *S. aureus *components has not yet been addressed. To determine the global impact of *S. aureus *components on primary human fibroblasts with respect to MMP expression, we exposed de-identified normal human dermal fibroblasts and synovial fibroblasts derived from de-identified patients with RA and osteoarthritis (OA) to whole cell lysate and culture supernatants (whole and fractionated) derived from *S. aureus *wild-type and mutant strains that induce less severe SA in murine models.

## Materials and methods

### Bacterial strains

*S. aureus *strain isolated from a patient with SA was obtained from American Type Culture Collection (ATCC) (Manassas, VA, USA). A de-identified clinical isolate (U1) and mutants lacking staphylococcal accessory gene regulator (U155: *Sar*^-/-^) and accessory gene regulator (U929: *Agr*^-/-^) and a strain lacking both *Sar A *and *Agr *(*Sar-Agr*^-/-^) derived from that clinical isolate were obtained from M. Smeltzer and were used in this study. The U155 strain was grown in the presence of tetracycline (5 μg/ml); U929 was grown in presence of kanamycin (50 μg/ml) and neomycin (50 μg/ml); and U930 was grown in the presence of tetracycline (5 μg/ml), 50 μg/ml kanamycin, and 50 μg/ml neomycin/ml for selection of the respective mutants. Strains grown in the presence of antibiotics were centrifuged, washed, and resuspended in Dulbecco's modified Eagle's medium (DMEM)/F-12 medium for inoculation in order to remove the antibiotics.

### Preparation of whole and fractionated bacterial culture supernatants and bacterial cell lysates

To collect supernatants and bacterial cell pellets for experiments, bacterial strains (isogenic parent strain and mutants strains) were grown in DMEM/F-12 containing 2% fetal bovine serum (FBS) without any antibiotics. Supernatants and cell pellets were obtained by centrifugation from 12-hour bacterial cultures. The supernatants were sterilized through 0.22-μm filters to ensure that they were free of any bacteria. The cell pellets were treated with lysostaphin (20 U/ml) for 20 minutes at 37°C followed by repeated freezing and thawing. The lysates were clarified by centrifugation at 12,000 *g *for 20 minutes and were filtered through 0.22-μm filters. The ATCC strain was also grown in the presence of 5 and 15 ng/ml recombinant human rhIL-1β (R&D Systems, Inc., Minneapolis, MN, USA). The cell lysates were prepared as described above. Total protein concentrations were measured by the calorimetric method (Bio-Rad, Hercules, CA, USA) in accordance with the manufacturer's instructions. The culture supernatants from the ATCC strain were fractionated into <30, 30 to 50, and >50 kDa molecular-weight fractions using respective Centricon filter centrifugation.

### Fibroblast cultures

Dermal fibroblasts from de-identified normal volunteers and synovial fibroblasts from de-identified RA patients and OA patients were maintained in DMEM/F-12 containing 10% FBS, 100 U/ml penicillin, and 100 μg of streptomycin. All the fibroblast cell lines were from a cell culture bank established by A. Postlethwaite in accordance with the full approval of the institutional review board of the University Of Tennessee Health Science Center (Memphis, TN, USA).

### Treatment of fibroblasts with *S. aureus *supernatants, lysates, and rhIL-1β/rhTNF-α

For studies measuring MMP production, 10^5 ^fibroblasts harvested by trypsinization were added to each well of 24-well tissue-culture plates (Falcon; Falcon Plastics, Inc., Washington, PA, USA). Three days later, confluent monolayers of fibroblasts were treated with phosphate-buffered saline, 25 μg of total proteins from bacterial cell lysates, 25 μg of total proteins from culture supernatants, and 15 μg of protein from each fraction of culture supernatant per well (only from ATCC strain). Fibroblasts were cultured in an incubator containing a humidified atmosphere containing 5% CO_2 _at 37°C. Fibroblasts were cultured for 8 hours for RNA analysis and 48 hours for protein analysis. Fibroblasts were also treated with a combination of 10 μg each of rhIL-1β/TNF-α for 8 hours and 48 hours. For mRNA analysis, cells were harvested after 8 hours of respective treatments, and total RNA was isolated using TRI-Reagent (Sigma-Aldrich, St. Louis, MO, USA) followed by isopropanol precipitation. The fibroblast culture supernatants were collected 48 hours after respective treatments and centrifuged to remove any cell debris. All samples were stored at -80°C until analyzed.

### Fractionation of *S. aureus *culture supernatants

Culture supernatants from *S. aureus *were purified using the Amicon Centricon filter device from Millipore Corporation (Billerica, MA, USA). Using this device, an approximately 2.0-ml volume was concentrated into an approximately 30-μl volume. Using the 10,000, 30,000, and 50,000 kDa cutoff filter devices, we fractionated the whole culture supernatants to <30, 30 to 50, and >50 kDa fractions.

### Assay for MMP and tissue inhibitor of metalloproteinases mRNAs

Total RNA was reverse-transcribed into cDNAs by using avian myeloblastoid virus (AMV) RTase and Oligo dT primers. Qualitative profiling of multiple MMP mRNAs was performed using a Multi-MMP-mRNA kit from SuperArray Bioscience Corporation (Frederick, MD, USA) in accordance with the manufacturer's protocol. Relative quantification of MMP mRNAs was performed using SYBR green real-time polymerase chain reaction (PCR) on the cDNAs obtained. Assays for MMP-1, -2, -3, -7, -8, -9, -10, -11, -12, -13, and -14 were performed. Gene-specific oligonucleotide primers for tissue inhibitor of metalloproteinases (TIMP)-1, TIMP-2, and TIMP-3 were synthesized at Integrated DNA Technologies (Coralville, IA, USA). The messages of TIMP mRNAs were quantified using SYBR green real-time reverse transcription-PCR (RT-PCR). The message levels were expressed as ratios of the threshold cycle values of respective MMP or TIMP messages to those of the housekeeping gene glyceraldehyde phosphate dehydrogenase (*GAPDH*).

### Assays for MMP protein

Multiple MMP proteins were determined using an MMP protein array kit from RayBiotech, Inc. (Norcross, GA, USA) strictly following the manufacturer's instructions. Briefly, the 100 μl of culture supernatants was applied to the membrane with arrayed antibodies. After blocking the free spaces on the membrane, a cocktail of biotin-labeled antibodies was added and the membranes were incubated for 2 hours at ambient temperature. After repeated washing, horseradish peroxidase-conjugated streptavidin was added onto the membrane and incubated for 2 hours at ambient temperature. After extensive washing, the detection buffer was added and the signals were detected by capturing the enhanced chemiluminiscence onto a Kodak x-omat AR film (Eastman Kodak, Rochester, NY, USA). The film was photographed and scanned for documentation.

### Semiquantitative profiling of mRNAs of MAPK family

A human MAPK gene family multigene-12 RT-PCR profiling kit (Superarray Bioscience Corporation) was used for the qualitative assessment of extracellular signal regulated kinase (ERK) 2/MAPK2, ERK1, MAPK4, ERK3, ERK5, c-jun N-terminal kinase (JNK) 1, JNK2, JNK3, p38b MAPK, p38g MAPK, and p38delta MAPK mRNA in fibroblasts in response to *S. aureus *culture supernatant and cell lysate. Total RNA was reverse-transcribed into cDNAs using AMV RTase and Oligo dT primers, and the messages were amplified using the primer sets supplied by the manufacturer. The expression level of the housekeeping gene *GAPDH *in each sample was used to assess the qualitative differences in respective message levels between samples. The experiments were repeated three times and each time the assays were set up in duplicate. The PCR products were analyzed on a 2% agarose gel and were stained with SYBR green. The intensities of the bands were estimated by densitometric scanning software from Alpha Innotech Corporation (San Leandro, CA, USA). The values expressed are ratios of the densities of the MAPK genes to those of the housekeeping gene *GAPDH*.

### Cell-based enzyme-linked immunosorbent assay for tyrosine phosphorylation

A cell-based phosphotyrosine enzyme-linked immunosorbent assay (ELISA) kit from RayBiotech, Inc., was used to quantitate tyrosine phosphorylation in human dermal fibroblasts in response to *S. aureus *components and IL-1/TNF. Approximately 30,000 cells were seeded into each well in a 96-well plate. Cells were incubated at 37°C, 5% CO_2 _overnight. The cells were then exposed to *S. aureus *cell lysate (25 μg/ml), *S. aureus *(ATCC) culture supernatant (25 μg/ml), or 10 ng/ml each of rhIL-1β and rhTNF-α for 30 minutes. The medium was removed from the wells, and the cells were treated with the fixing solution followed by quenching solution. The fixed, quenched cells were treated with blocking solution for 3 hours at ambient temperature, and after washing the cells were exposed to anti-phosphotyrosine-horseradish peroxidase for 1 hour followed by washing and the addition of one-step substrate solution. The plates were incubated in the substrate solution for 30 minutes, the color reaction was stopped, and the optical densities were read at 450 nm. The experiments were repeated three times and each time the experiments were run in triplicates.

### Statistical analysis

Each treated sample was compared with the untreated sample using Student's test. Sigma Stat (Systat Software, Inc., San Jose, CA, USA) program was used for statistical computation, and Sigma Plot (Systat Software, Inc.) was used to create graphs. A *p *value of less than 0.05 was considered significant.

## Results

### Induction of multiple MMP proteins by *S. aureus *in human dermal fibroblasts

**Figure 1:**
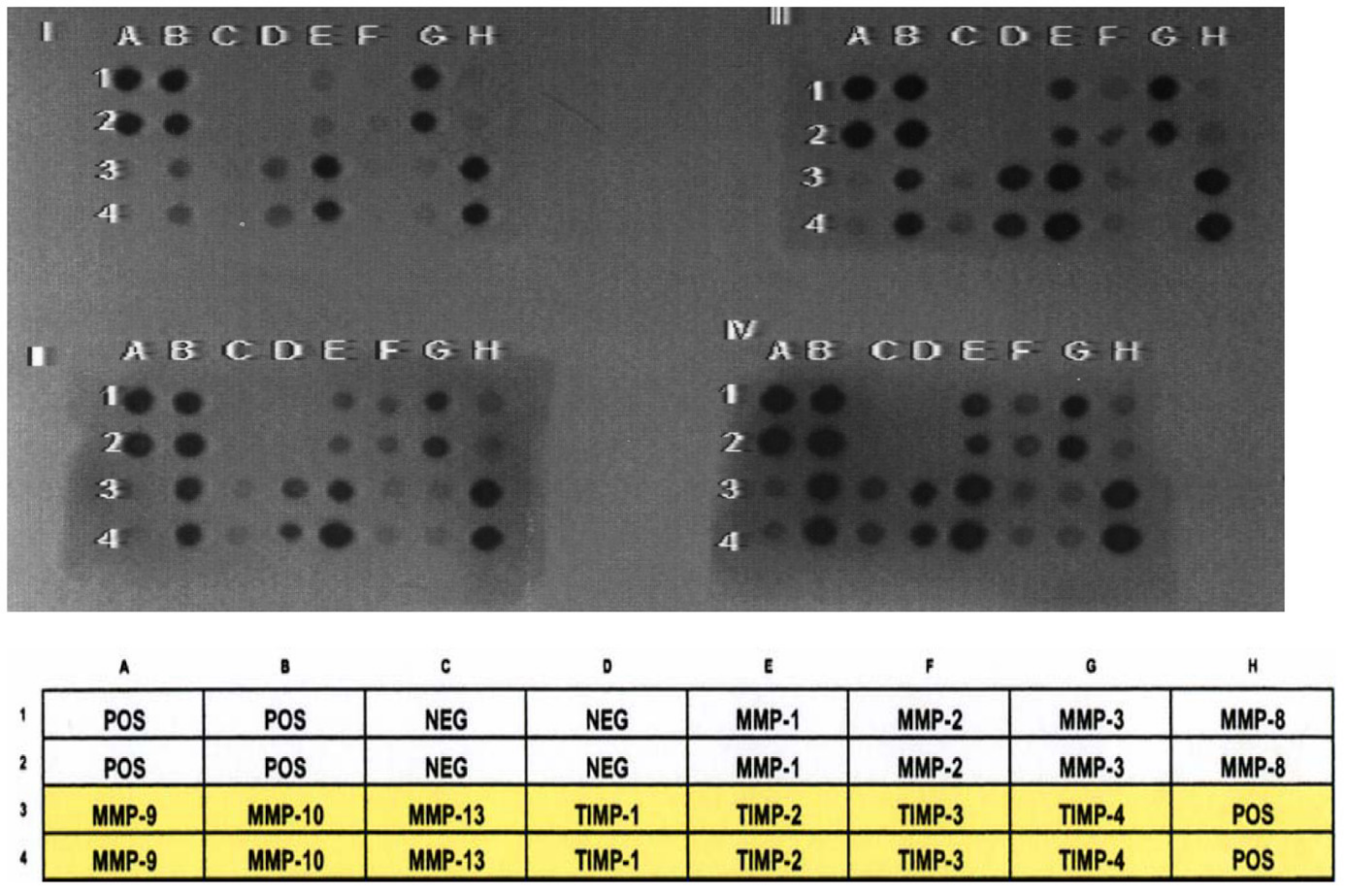
Induction of multiple matrix metalloproteinase (MMP) protein by *Staphylococcus aureus *in human dermal fibroblasts. Confluent monolayers of human primary dermal fibroblasts were exposed to **(I) **phosphate-buffered saline, **(II) **25 μg/well of total protein from clarified whole cell lysates of *S. aureus *cells, **(III) **25 μg of total protein from the filtered culture supernatant of *S. aureus*, and **(IV) **treated with 10 ng/m each of interleukin-1β/tumor necrosis factor-α (IL-1β/TNF-α) for 48 hours. The fibroblast culture supernatants were harvested and the MMPs and tissue inhibitors of metalloproteinases (TIMPs) were detected using the MMP-Array kit from RayBiotech, Inc. (Norcross, GA, USA). Culture supernatant and cell lysate from *S. aureus *induced the expression of MMP-1, MMP-2, MMP-10, and MMP-13. A similar pattern was observed in IL-1β/TNF-α-treated fibroblasts, which were slightly more intense. There was upregulation of both TIMP-1 and TIMP-2 as well.

Culture supernatant and cell lysate from *S. aureus *(ATCC) induced the expression of immunoreactive proteins of MMP-1, MMP-2, MMP-10, and MMP-13 in dermal fibroblasts (Figure 1). Upregulation of TIMP-1 and TIMP-2 was also noted in *S. aureus *culture supernatant and cell lysate-treated fibroblasts. There were no notable changes in the expression levels of other MMP proteins (MMP-8 and MMP-9) in the cells in response to treatment. The expression pattern and level of expression were similar in *S. aureus *components and IL-1β/TNF-α-treated fibroblasts.

### Induction of multiple MMP mRNAs by *S. aureus *in human dermal and synovial fibroblasts

**Figure 2:**
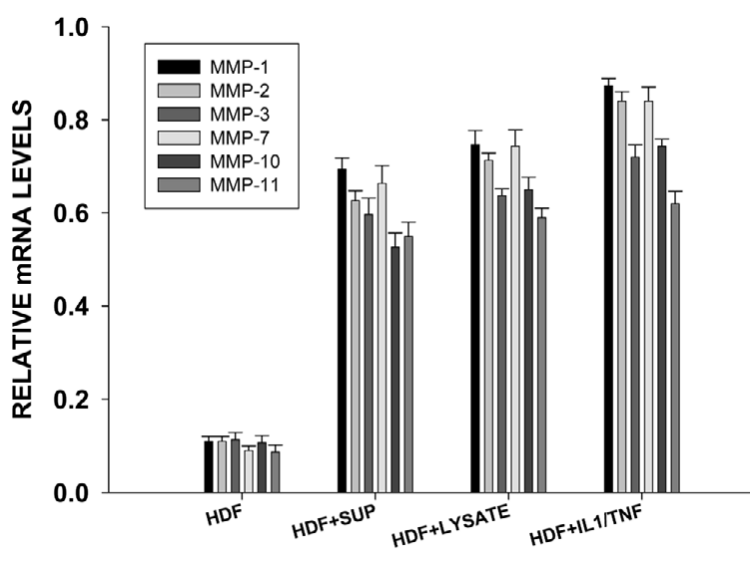
Induction of multiple matrix metalloproteinase (MMP) mRNAs in human dermal fibroblasts (HDF) by *Staphylococcus aureus*. Confluent monolayers of fibroblasts were exposed to 25 μg/well of total protein from culture supernatants (SUP), 25 μg/well of total protein from bacterial cell lysate, or 10 ng/ml each of rhTNF-α and rhIL-1β for 8 hours. Total RNA was isolated and reverse-transcribed. The cDNA was amplified and quantified using SYBR green real-time polymerase chain reaction. All the MMPs tested were expressed in dermal fibroblasts exposed to *S. aureus *components. Both culture supernatants and cell lysate from *S. aureus *significantly enhanced the expression of multiple MMPs (namely, MMP-1, -2, -3, -7, -10, and -11; *p *< 0.05). The dermal fibroblasts responded similarly to a combination of rhTNF-α and rhIL-1β in terms of multiple MMP mRNA expression. IL, interleukin; TNF, tumor necrosis factor.

**Figure 3:**
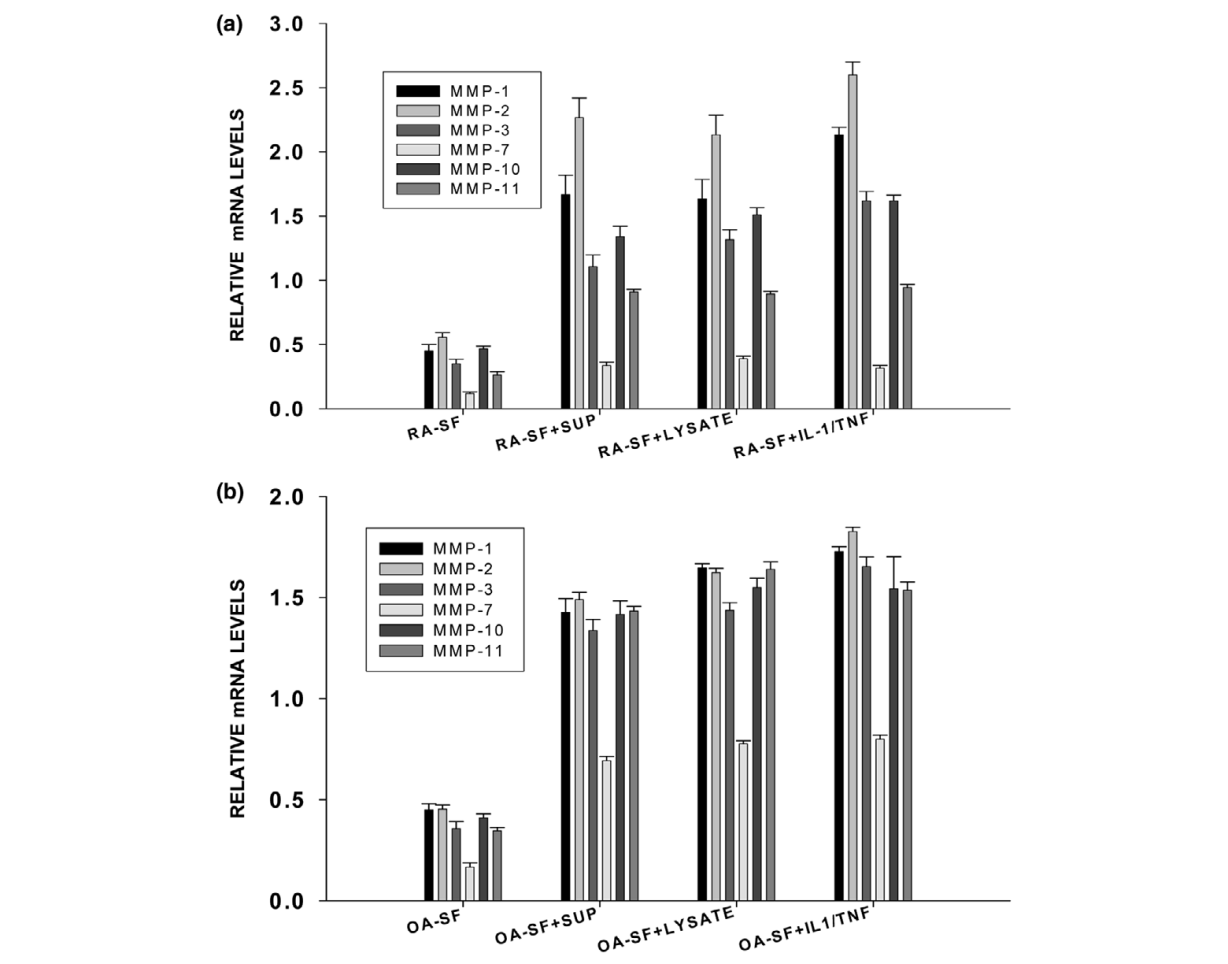
Induction of multiple matrix metalloproteinase (MMP) mRNAs in human synovial fibroblasts (SFs) by *Staphylococcus aureus*. MMP mRNA expression profile in confluent monolayers of SFs obtained from de-identified **(a) **rheumatoid arthritis (RA) and **(b) **osteoarthritis (OA) patients treated with culture supernatants (SUP) (25 μg of total protein/well) and cell lysates (25 μg of total protein/well) were determined using SYBR green real-time reverse transcription-polymerase chain reaction. Unlike untreated dermal fibroblasts, untreated SFs from RA and OA patients had higher basal multiple MMP mRNA expression. Similar to the dermal fibroblasts, most MMP mRNAs tested were elevated in RA and OA fibroblasts in response to *S. aureus *cell lysate and culture supernatants. Levels of significantly elevated MMPs (MMP-1, -2, -3, -7, -10, and -11; *p *< 0.05) are shown in **(a) **and **(b)**. The MMP mRNA expression pattern in response to IL-1β/TNF-α and *S. aureus *lysate was similar in RA and OA fibroblasts. IL, interleukin; TNF, tumor necrosis factor.

Multiple MMP mRNA profile in dermal and synovial fibroblasts in response to *S. aureus *components was determined by SYBR green real-time PCR. Culture supernatants and cell lysate from *S. aureus *significantly enhanced the expression of multiple MMP mRNAs (namely, MMP-1, -2, -3, -7, -10, and -11; *p *< 0.05). As in the case of MMP protein expression pattern, the response of the fibroblasts in terms of MMP mRNA expression was similar in *S. aureus *component-treated and rhTNF-α- and rhIL-1β-treated fibroblasts (Figure 2). Unlike untreated dermal fibroblasts (Figure 2), untreated synovial fibroblasts from patients with RA (Figure 3a) and OA (Figure 3b) had higher basal multiple MMP mRNA expression, indicating an activated status of the synovial fibroblasts from a pathological site. Similar to the dermal fibroblasts, most MMP mRNAs tested were elevated in RA and OA fibroblasts in response to *S. aureus *components. Levels of significantly elevated MMPs (MMP-1, -2, -3, -7, -10, and -11; *p *< 0.05) are shown in Figure 3a,b. The MMP mRNA expression pattern in response to IL-1β/TNF-α and *S. aureus *lysate was similar in RA and OA fibroblasts (Figure 3a,b) as well. Interestingly, no significant differences were noted in MMP-13 mRNA levels between the treated and untreated fibroblasts. All other MMPs tested were expressed at very low levels, could not be quantified, and hence were not included in the graph.

Two more dermal fibroblast lines, RASF and OASF cell lines, were tested for multiple MMP mRNA expression profile upon exposure to *S. aureus *culture supernatants and bacterial cell lysates. Essentially the same profile as described above was obtained from the additional cell lines (data not shown). Because fibroblasts are heterogeneous in terms of their origin and some of their features, it is likely that fibroblasts from different sources may respond slightly differently in terms of MMP expression.

### Potentiation of MMP protein expression in human fibroblasts by *S. aureus *grown in presence of rhIL-1β

**Figure 4:**
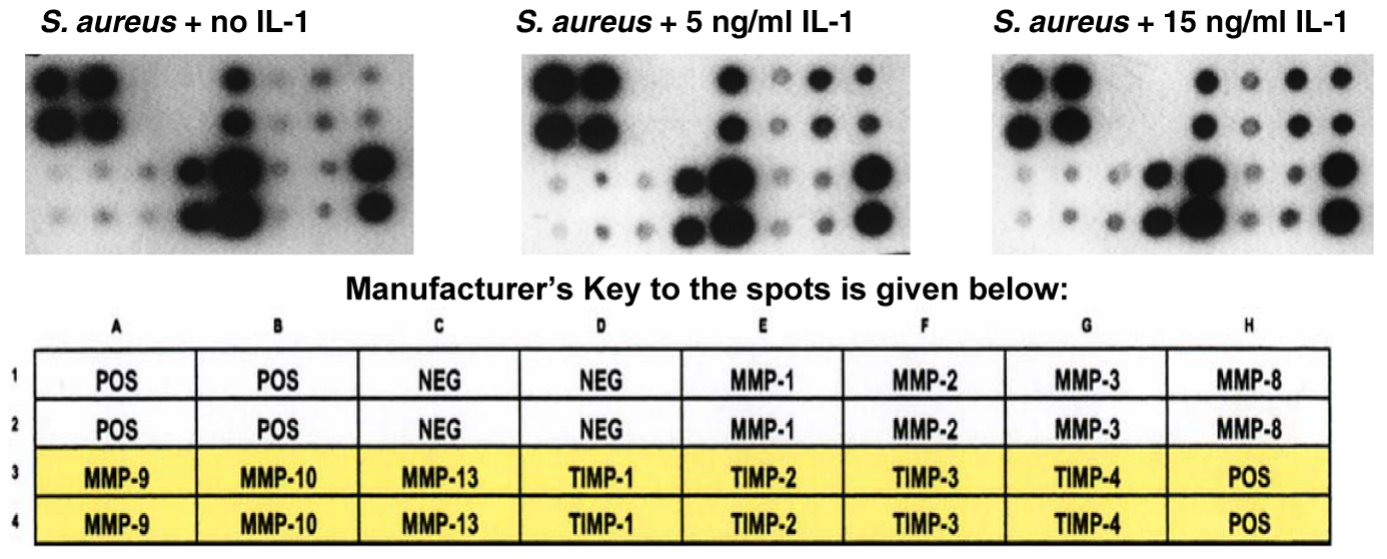
Potentiation of matrix metalloproteinase (MMP) protein expression in human fibroblasts by *Staphylococcus aureus *grown in the presence of recombinant human interleukin (rhIL)-1β. The cell pellets from *S. aureus *grown in the presence or absence of rhIL-1β were washed repeatedly to prepare cell lysate. Enzyme-linked immunosorbent assay (R&D Systems, Inc., Minneapolis, MN, USA) was carried out to make sure that the bacterial cell lysate was devoid of any traces of rhIL-1β. Confluent monolayers of human dermal fibroblasts were exposed to 25 μg/well of IL-1β-free cell lysate obtained from *S. aureus *for 48 hours. The supernatants were collected and expression of multiple MMP protein was assessed using multi-MMP-Array kit from RayBiotech, Inc. (Norcross, GA, USA) as described in the text. The data presented show that MMP-2, -3, and 8 are relatively more expressed in fibroblasts treated with *S. aureus *cell lysate obtained from strain grown in the presence of 5 and 15 ng/ml rhIL-1β. Tissue inhibitor of metalloproteinases (TIMP)-4 expression was also slightly enhanced in fibroblasts treated with *S. aureus *lysate obtained from strain treated with 15 ng/ml IL-1β.

We have observed significant changes in gene expression in *S. aureus *grown in the presence of rhIL-1β (manuscript submitted). To test whether *S. aureus *grown in the presence of rhIL-1β would have any impact on MMP expression, dermal fibroblasts were exposed to 25 μg/ml per well bacterial cell lysate obtained from *S. aureus *grown in the presence of 5 or 15 ng/ml rhIL-1β. The supernatants were collected and expression of multiple MMP protein was assessed by multi-MMP-Array kit from RayBiotech, Inc., as described previously. The data presented in Figure 4 show that production of MMP-2, -3, and -8 is greater in fibroblasts treated with cell lysate obtained from the *S. aureus *strain grown in the presence of rhIL-1β. TIMP-4 expression was also slightly enhanced in fibroblasts treated with lysate obtained from the *S. aureus *grown in the presence of 15 ng/ml IL-1β.

### Induction of MMP mRNA in human dermal fibroblasts by fractionated culture supernatants from *S. aureus*

**Figure 5:**
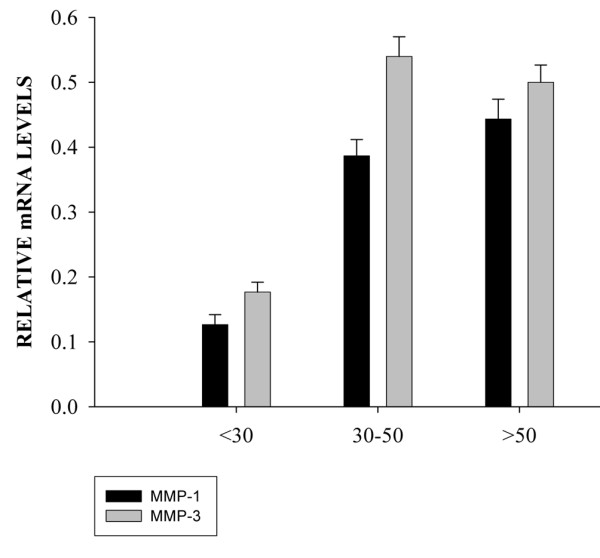
Induction of matrix metalloproteinase (MMP)-1 and MMP-3 mRNA in response to *Staphylococcus aureus *components separated by molecular-weight difference. Fractionated *S. aureus *culture supernatant (15 μg/ml per well) obtained by centrifuging through Centricon filters with molecular-weight cutoff points <30 kDa, 30 to 50 kDa, and >50 kDa was added to confluent monolayers of dermal fibroblasts. Total RNA was isolated after 8 hours. The mRNA levels of MMP-1 and MMP-3 were measured by SYBR green real-time reverse transcription-polymerase chain reaction. The active components were mostly in the 30 to 50 and >50 kDa molecular-weight range as evidenced by significantly elevated expression of MMP-1 and MMP-3 by Centricon fractions 30 to 50 and >50 kDa (*p *< 0.05; 30 to 50 kDa and >50 kDa fractions were compared with <30 kDa fractions).

The MMP-inducing active components in the culture supernatants were mostly in the 30 to 50 and >50 kDa molecular-weight range as evidenced by significantly elevated expression of MMP-1 and MMP-3 by Centricon fractions 30 to 50 and >50 kDa in dermal fibroblasts (Figure 5; *p *< 0.05). Although the fractions are not identified beyond their molecular weight, this does rule out some of the already-characterized low-molecular-weight extracellular (secreted) products of *S. aureus*.

### MMP mRNA induction by *Sar*, *Agr*, and *Sar/Agr *mutants of *S. aureus*

**Figure 6:**
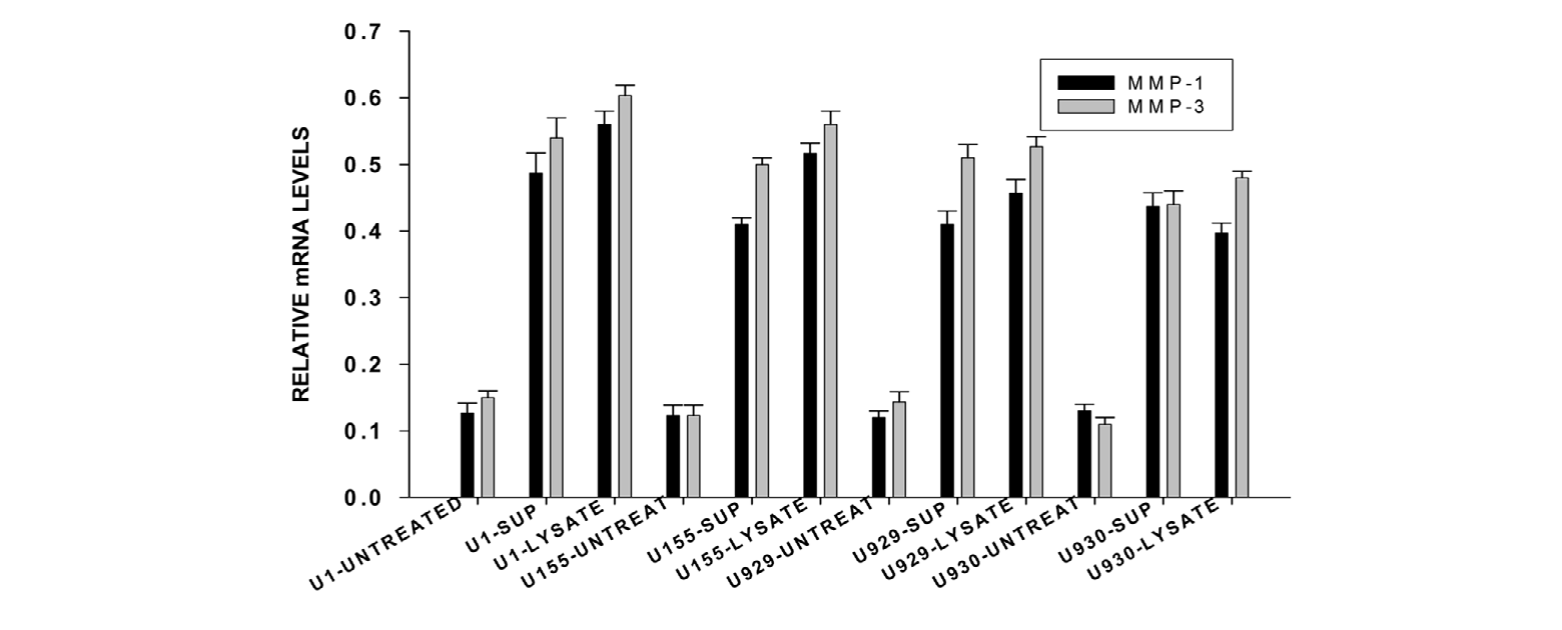
Matrix metalloproteinase (MMP) mRNA induction by staphylococcal accessory regulator (*Sar*), accessory gene regulator (*Agr*), and *Sar/Agr *mutants of *Staphylococcus aureus*. MMP-1 and MMP-3 mRNAs in confluent monolayers of human dermal fibroblasts exposed to 25 μg of total protein/well per milliliter from cell lysate and 25 μg/well per milliliter of total protein from culture supernatants (SUP) of parent (U1) mutant strains (U155 *Sar*^-/-^; U929 *Agr*^-/-^; U930 *Sar/Agr*^-/-^) were measured by SYBR green real-time reverse transcription-polymerase chain reaction 8 hours after exposure. No significant differences in mRNA levels of MMP-1 and MMP-3 induced by the mutants and their isogenic parent strain were observed.

Blevins and colleagues [19] have shown that *S. aureus *strains lacking the regulatory loci *Sar *or *Agr *result in less severe SA and osteomyelitis in murine models of these diseases. We therefore tested the ability of cell lysates and culture supernatants obtained from these mutants and their isogenic parent strain to induce MMP-1 and MMP-3 mRNAs in human dermal fibroblasts. The mutants and isogenic strains enhanced MMP-1 and MMP-3 production by fibroblasts to a similar degree (Figure 6).

### Induction of TIMP mRNA expression in human fibroblasts by *S. aureus *wild-type and *Sar/Agr *mutants

**Figure 7:**
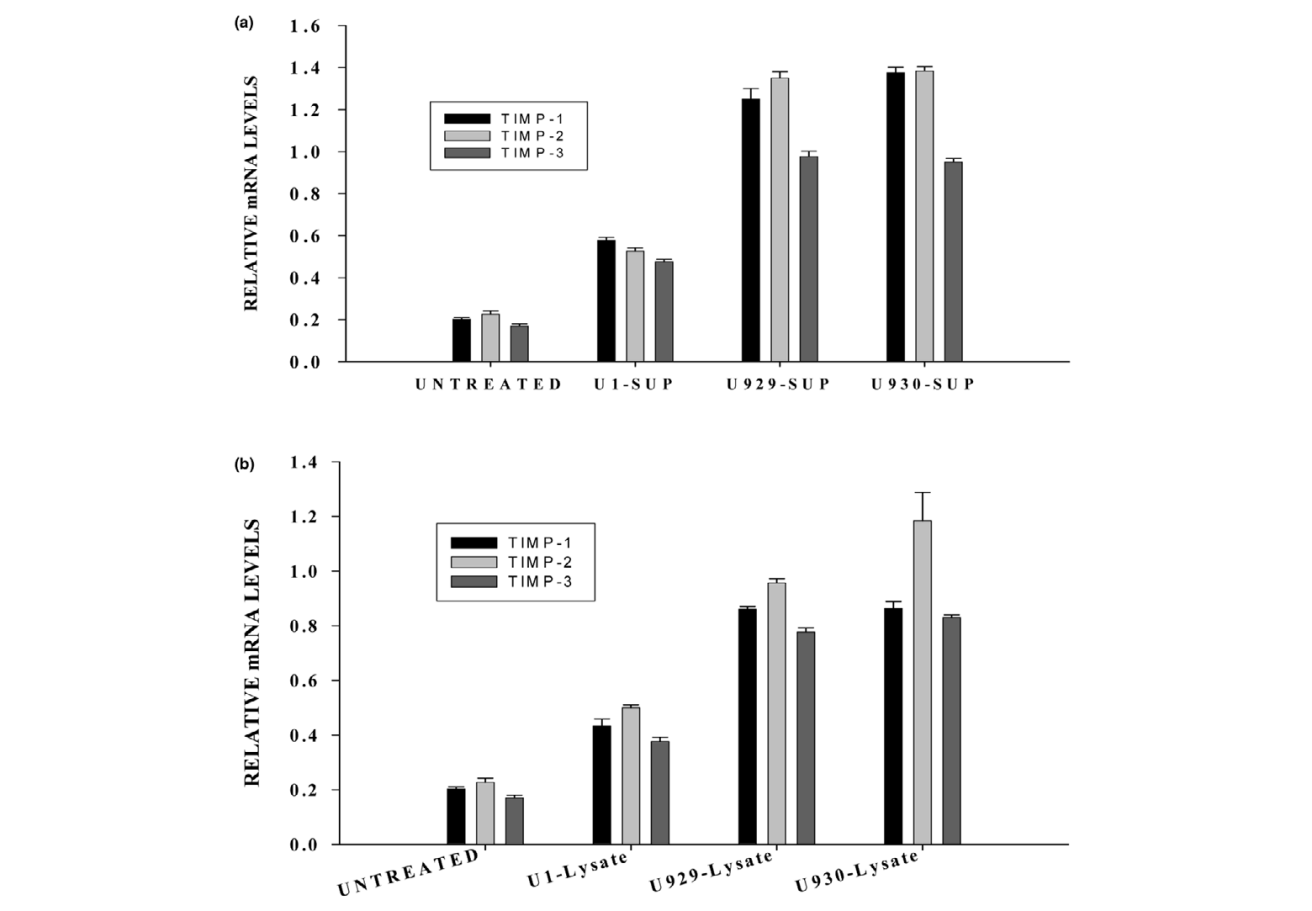
Enhanced expression of tissue inhibitor of metalloproteinases (TIMP) mRNAs in human dermal fibroblasts treated with culture supernatants **(a) **and whole bacterial cell lysates **(b) **obtained from *Sar A*^-/- ^and *Sar A*^-/-^Agr^-/- ^mutants of *Staphylococcus aureus *and isogenic parent strain. Culture supernatants and cell lysates obtained from an *S. aureus *strain isolated from synovial fluid of a septic arthritis patient (obtained from American Type Culture Collection, Manassas, VA, USA), a clinical isolate (U1), its *Sar A *mutant (U929), and *Agr/Sar A *double-loci-deleted mutant (U930) (strains obtained from M. Smeltzer) were used. Equal amounts of total protein from all samples were added to confluent monolayers of fibroblast cultures. Total RNA was harvested from the cells 12 hours after exposure to the supernatants. The levels of TIMP-1, -2, and -3 mRNAs were estimated by real-time SYBR green reverse transcription-polymerase chain reaction. The values plotted represent the ratios of the threshold cycle values of TIMPs to that of the housekeeping gene *GAPDH *(glyceraldehyde phosphate dehydrogenase). Agr, accessory gene regulator; Sar, staphylococcal accessory regulator; SUP, culture supernatant.

TIMPs are members of the MMP gene family and play an important role in the overall availability of active MMPs. Hence, it is important to determine the TIMP expression profile of fibroblasts in response to *S. aureus *and *S. aureus *components. In our current study, we used culture supernatants obtained from an *S. aureus *strain isolated from synovial fluid of a patient with SA (ATCC), a clinical isolate (U1), and its *Agr/Sar A *double-loci-deleted mutant U930 (strains obtained from M. Smeltzer). The results presented in Figure 7a,b indicate a notably increased induction of TIMP-1, -2, and -3 mRNA by the *Agr/Sar A *deletion mutant (U930) of the isogenic parent wild-type strain (U1) and the ATCC strain isolated from the synovium of a patient with arthritis.

It may be speculated that the effective MMP available upon infection with *Agr/Sar *deletion mutant is likely to be less compared with the parent isogenic strain. However, further studies to examine expression of other MMPs as well as analysis to estimate enzymatically active MMPs by zymogram will be required to determine whether genes under the control of *Sar *or *Agr *have any effect on the expression of functional MMPs.

### MAPK gene expression in synovial fibroblasts from patients with RA and OA

**Figure 8:**
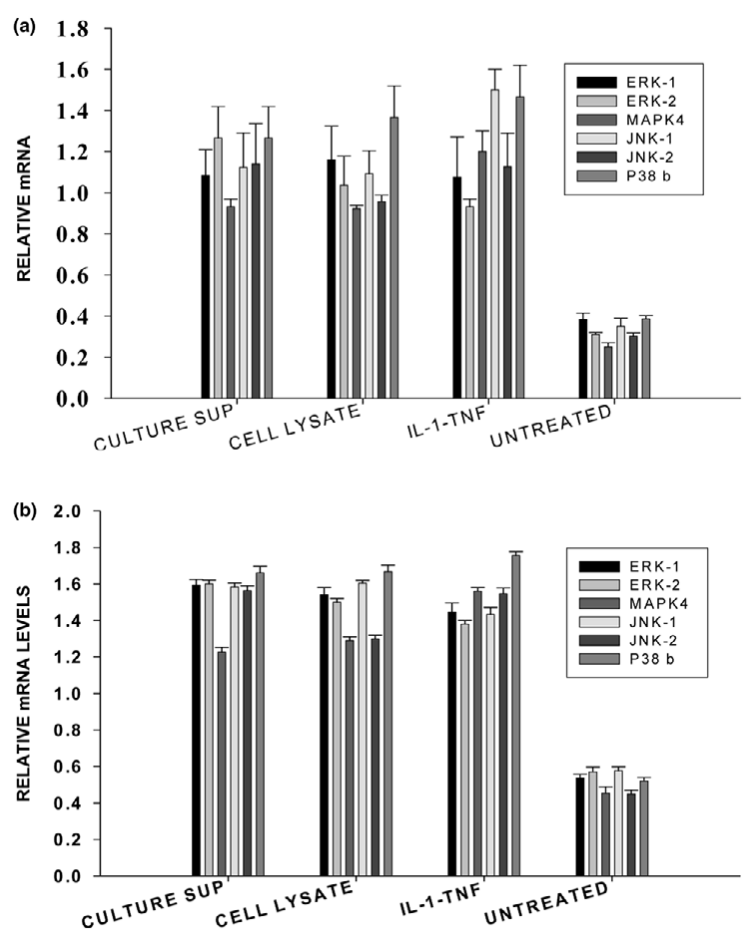
Mitogen-activated protein kinase (MAPK) family mRNA expression profile in human dermal fibroblasts **(a) **and human synovial fibroblasts **(b) **exposed to *Staphylococcus aureus *whole cell lysate and filtered culture supernatant. Confluent monolayers of de-identified human dermal fibroblasts from normal volunteer and synovial fibroblasts from a patient with rheumatoid arthritis were treated with 25 μg/ml per well of *S. aureus *whole lysate, filtered culture supernatant, and a combination of rhIL-1β and TNF-α (10 ng/ml each) for 8 hours. Cells were harvested and total cellular RNA was isolated and reverse-transcribed. The MAPK family gene expression was analyzed using the Human MAPK Gene Family I Multigene-12 reverse transcription-polymerase chain reaction (PCR) profiling kit. The semiquantitative values were generated by determining the ratios of the band intensities of respective PCR products to that of housekeeping gene *GAPDH *(glyceraldehyde phosphate dehydrogenase) in each sample. The experiments were repeated three times. The band densities were determined using three-dimensional densitometric scanning software from Alpha Innotech Corporation (San Leandro, CA, USA). Of the 11 tested Human MAPK Family I genes (ERK1, ERK2, MAPK2/4, ERK3, ERK5, JNK1, JNK2, JNK3, p38b MAPK, p38g MAPK, and p38delta), ERK1, ERK2, JNK1, JNK2, MAPK4, and p38b were elevated significantly in both dermal and synovial fibroblasts upon exposure to *S. aureus *components (*p *< 0.05 for individual MAPK gene family member when values from untreated fibroblasts and treated fibroblasts were compared). The response was similar in both cell lines in response to IL-1/TNF. ERK, extracellular signal regulated kinase; IL, interleukin; JNK, c-jun N-terminal kinase; SUP, filtered culture supernatant; TNF, tumor necrosis factor.

Members of the MAPK gene family (such as ERK1/2 and p38MAPK) are involved in the induction of MMPs through activation protein (AP-1) transcription factors. We therefore analyzed the mRNA expression levels of 12 members of the MAPK family using the MultiGene-12 RT-PCR profiling kit from Superarray Bioscience Corporation. Synovial fibroblasts obtained from patients with RA and OA were exposed to 25 μg of total proteins from bacterial culture supernatant or cell lysate, and total RNA was isolated 6 hours later, reverse-transcribed, and assayed for mRNA of 12 MAPK genes. Several of the MAPK family members were upregulated. The ratio between the intensities of each MAPK gene to that of *GAPDH *is depicted in Figure 7. Significant increases in ERK2, ERK1, MAPK4, JNK1, JNK2, p38b, and p38g were observed in dermal fibroblasts treated with *S. aureus *culture supernatant and cell lysate-treated compared with untreated fibroblasts (Figure 8a; *p *< 0.05) and in synovial fibroblast-treated compared with untreated fibroblasts (Figure 8b; *p *< 0.05). Similar increases in these MAPK gene family members were noted in IL-1β/TNF-α-treated fibroblasts (Figure 8a,b; *p *< 0.05).

### Tyrosine phosphorylation in human dermal fibroblasts exposed to *S. aureus *culture supernatant

**Figure 9:**
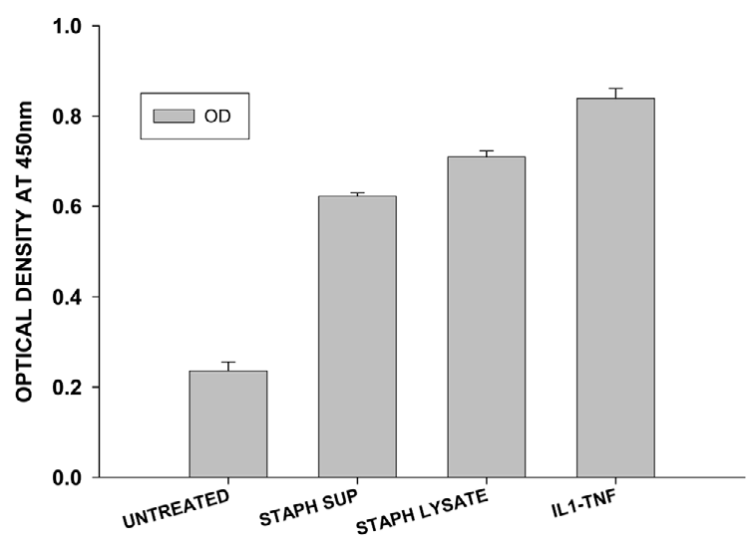
Tyrosine phosphorylation in human dermal fibroblasts exposed to *Staphylococcus aureus *culture supernatant. Using a cell-based enzyme-linked immunosorbent assay system (RayBiotech, Inc., Norcross, GA, USA), tyrosine phosphorylation was assessed in human dermal fibroblasts exposed to 25 μg of total protein/ml from filtered culture supernatant of *S. aureus*, 25 μg/ml total protein from *S. aureus *cell pellet lysate, and in fibroblasts treated with 10 ng/ml each of rhIL-1β and rhTNF-α. These assays were carried out in 96-well plates supplied by the manufacturer. The cells were exposed to various agents for 30 minutes before assaying for tyrosine phosphorylation. The assays were carried out in triplicate. There was significant increase in tyrosine phosphorylation in *S. aureus *culture supernatant-treated cells similar to IL-1β/TNF-α-treated cells compared with untreated fibroblasts (*p *< 0.05). IL, interleukin; OD, optical density; SUP, culture supernatant; TNF, tumor necrosis factor.

Using a cell-based ELISA system (RayBiotech, Inc.), tyrosine phosphorylation was assessed in human dermal fibroblasts after 30-minute exposure to 25 μg of total protein from filtered culture supernatant of *S. aureus *and in fibroblasts treated with 10 ng/ml each of rhIL-1β and rhTNF-α. There was a significant increase in phosphotyrosine in *S. aureus *culture supernatant-treated cells, similar to that observed in IL-1β/TNF-α-treated cells (Figure 9; *p *< 0.05).

Overall, our data indicate that *S. aureus *components induce multiple MMP expression in human dermal and synovial fibroblasts and that the response is similar to that induced by IL-1β/TNF-α. The expression pattern of MAPK gene expression also indicates the possibility of a signal transduction pathway akin to that induced by the inflammatory cytokine pathway. Our data also indicate that the virulence gene loci (namely, *Sar *and *Agr*) are not determinants of *S. aureus*-induced MMP mRNA expression.

## Discussion

We have shown that the culture supernatants and whole bacterial lysate from *S. aureus *induce multiple MMPs from human dermal and synovial fibroblasts. Several genes of the MAPK pathways were upregulated in treated fibroblasts, and phosphotyrosine proteins were significantly elevated. Using fractionated *S. aureus *culture supernatants, we have shown that the best MMP induction was by components that fall within the molecular-weight range of 30 to 50 kDa. Interestingly, culture supernatants and bacterial cell lysates obtained from *S. aureus *grown in the presence of rhIL-1β induced notably higher levels of MMPs compared with *S. aureus *grown in the absence of rhIL-1β. The overall spectrum of MMP induction by *S. aureus *components was similar to that elicited by a combination of IL-1β and TNF-α. Our *in vitro *MMP mRNA expression analysis showed that mutants lacking *Sar A *and *Agr *loci and their parent isogenic strain induced comparable levels of MMP mRNAs; however, the mutant strains induced notably higher levels of TIMP-1, -2, and -3 mRNAs in human fibroblasts. To our knowledge, this is the first report on multiple MMP/TIMP induction by fractionated *S. aureus *culture supernatants and whole bacterial cell lysates in human dermal and synovial fibroblasts.

SA is the most commonly reported bacterial complication of RA. The risk is highest in severe, longstanding, seropositive disease. The clinical presentation of joint infection is frequently atypical, and in 25% of cases, the infection is polyarticular. *S. aureus *is the most common causative organism [20]. Staphylococcal infections can be hard to eradicate from RA joints and often surgery is required [21]. TNF-α plays an important role in the host defense against infection. Inhibition of its activity could therefore be anticipated to augment the risk of infection in patients with RA. The reasons for the predominance of *S. aureus *in SA and the mechanisms of pathogenecity are not yet fully understood. The synovium of patients with RA is rich in IL-1β. We have previously shown that *S. aureus *can bind to IL-1β and use it as a growth factor [22]. A recent report by McLaughlin and Hoogewerf [23] showed that the growth and replication of *S. aureus *in a biofilm are significantly increased by the addition of rhIL-1β. We have also observed that rhIL-1 can modulate the gene expression in *S. aureus *including the bicomponent leukotoxins and some of the surface adhesion molecules collectively called MSCRAMMs (microbial surface components recognizing adhesive matrix molecules) in addition to some of the genes in the pathogenecity island of *S. aureus *(Kanangat and colleagues, manuscript submitted). We speculate that the IL-1-rich synovial milieu might potentially contribute to the increased frequency of *S. aureus *in patients with RA-SA and that the host-derived MMPs induced by *S. aureus *might accelerate the pathogenesis of SA.

Our data on the induction of MMPs by *S. aureus *culture supernatants and cell lysates compares well with the previous report by Williams and colleagues [16], who demonstrated MMP-1 and -3 expression by articular cartilage upon exposure to purified culture supernatant from *S. aureus*. We have extended this observation by showing expression of a wide range of MMPs, including MMP-7 (with a proven *in vivo *association with *S. aureus*-induced SA), by human synovial as well as dermal fibroblasts in response to *S. aureus *components. The profile was similar to that induced by a combination of IL-1/TNF, which might indicate the involvement of an inflammatory cytokine-mediated pathway in the observed induction of MMPs by *S. aureus*.

*S. aureus *culture supernatants and cell lysates have a wide variety of proteins, and identification of the components that are actually responsible for inducing the MMP induction is essential to determine the mechanisms of induction as well as to rationally design intervening agents against bacterial products. Toward this we end, we have narrowed down the possible candidates to molecular-weight groups of the range of >30 to 50 based on our experiments using Centricon filtration of the culture supernatants. Because the molecular weight of the chemically purified PGN used in previous studies is not known, we are not in a position to determine whether PGN is included in the stated molecular-weight range. At this time, we have not identified the components beyond the molecular level; nevertheless, this rules out the possibility of some of the recently described low-molecular-weight proteins such as the 19-kDa extracellular fibrinogen-binding protein that inhibits complement activation [24]. Certain complement components have been reported to activate MMPs [25]. The results of the fractionated supernatants also tentatively rule out the possibility of the exotoxin akin to the toxic shock syndrome protein described by Ren and colleagues [26] and the enterotoxin H described by Su and Wong [27]. Although speculative at this juncture, it is possible that the active components in the 30 to 50 kDa could potentially be the novel 38.5-kDa protein named extracellular matrix-binding protein described by Hussain and colleagues [28].

Joint destruction by *S. aureus *is very rapid if not treated appropriately. Although direct erosion of the joint architecture by *S. aureus *proteases/toxins cannot be completely ruled out, continued degradation of extracellular matrix component and the joint architecture even after clearing the infection and debris from the joint cavity indicates the possibility of host-derived proteases in causing joint pathology. Previous studies have shown the release of active MMP-1 and MMP-3 by human articular cartilage upon exposure to sterile purified *S. aureus *culture medium [16]. The enzymatic profile was similar to that induced by IL-1. The authors concluded that the collagenase and stromelysin released by articular cartilage could contribute to extensive destruction of human cartilage in SA. The exoproteases of *S. aureus *have been proposed as virulence factors during *S. aureus *infections. Calander and colleagues [29], using wild-type *S. aureus *strain 8325-4 and its mutants lacking aureolysin, serine protease, and cysteine protease, demonstrated in a murine SA model that inactivation of the exoprotease genes did not affect the frequency or the severity of joint pathology. Intra-articular injection of PGN into murine joints triggered arthritis in a dose-dependent manner [17]. A single injection of this compound caused massive infiltration of macrophages and polymorphonuclear cells with signs of cartilage and/or bone destruction, lasting for at least 14 days, indicating that PGN exerts a central role in joint inflammation triggered by *S. aureus *[17]. The significance of MMP-7 expression in SA was examined by Gjertsson and colleagues [30] using MMP-7-deficient mice and congeneic controls. These mice were inoculated (intravenously) with an arthritogenic dose of *S. aureus *LS-1, and the mice deficient for MMP-7 developed significantly less-severe arthritis both clinically and histologically despite significantly increased numbers of live bacteria within the internal organs. Interestingly, *in vitro *responses to staphylococcal antigens and superantigens were not different between MMP-7^+/+ ^and MMP-7^-/- ^mice in terms of cytokine production. MMP-7 facilitates migration of both macrophages and neutrophils, and the authors therefore conclude that modulation of SA by MMP-7 might be due to changes in peripheral leukocyte distribution. Also, studies by Wang and colleagues [31] have shown that addition of PGN to whole human blood resulted in enhanced levels of MMP-9 within 1 hour and significant enhancement of MMP-9 secretion from the neutrophils was obvious within 30 minutes of incubation with *S. aureus *PGN.

Host-cell MMPs are necessary for efficient clearance of infection by accelerating the recruitment of effector cells to kill pathogen and to resolve inflammation and for subsequent tissue remodeling [9]. However, excessive MMPs after infection and inflammation may cause tissue damage leading to immunopathology. MMPs are secreted by both inflammatory and connective tissue cells such as fibroblasts, fibroblast-related cells, chondrocytes, neutrophils, and monocytes/macrophages in response to both infectious assaults and inflammatory cytokines such as TNF-α and IL-1β [32,33]. The regulation of MMP secretion is dependent on the cell type and stimulus. Signal transduction pathways that involve the MAPK and prostaglandin E2/cyclic AMP pathways are considered to be crucial, although the involvement of other pathways cannot be ruled out [34,35]. The transcription factors c-jun (cellular homologue of viral oncogene jun) and c-fos (cellular homologue of FBJ murine sarcoma virus oncogene), members of the AP-1 family, are involved in the transcription of MMP-1 and these AP-1 factors are induced through MAPK signal transduction. We determined the mRNA levels of some of the MAPK family members in synovial fibroblasts from patients with RA or OA treated with *S. aureus *and lysate culture supernatant or IL-1β/TNFα. mRNAs for several of the MAPK family members were upregulated by *S. aureus *lysates and culture supernatants similar to those treated with IL-1β/TNF-α. Also, the overall phosphotyrosine expression was enhanced in fibroblasts treated with *S. aureus *components. The increase in phosphotyrosine in fibroblasts treated with *S. aureus *components or IL-1β/TNFα was comparable.

The importance of the proinflammatory cytokines TNF-α and lymphotoxin (LT)-α in an experimental model of *S. aureus *sepsis and arthritis was examined by Hultgren and colleagues [36]. Using TNF-α/LT-α-double-deficient mice, they showed that in mice inoculated intravenously with a toxic shock syndrome toxin-1-producing *S. aureus *strain LS-1, mortality in TNF-α/LT-α-deficient mice was 67%, with no mortality in the controls. Those results correlated with a significantly decreased phagocytosis *in vitro *and inefficient bacterial clearance *in vivo *in mice lacking the capacity to produce TNF-α/LT-α. Infection of mice with a lower dose of staphylococci resulted in an overall low mortality, but the frequency of arthritis was significantly higher in the wild-type group compared with the TNF-α/LT-α-deficient mice (40% versus 13%). Synovitis and erosivity were lower in TNF-α/LT-α-deficient mice compared with wild-type controls. This study demonstrates the detrimental role of TNF-α/LT-α as mediators of the inflammatory response in *S. aureus *arthritis. TNF-α is a potent inducer of several types of MMPs.

Although IL-1 or TNF induced by *S. aureus *could very well be contributing to the joint destruction (either through induction of MMPs or through other degradative pathways), studies by Kimura and colleagues [37] showed that blocking TNF and IL-1 does not significantly prevent the late-stage destruction of joint architecture in arthritis induced by *S. aureus*. In the murine heat-killed *S. aureus*-induced arthritis model, TNF-α and IL-1β peaked at 2 and 24 hours after the injection of heat-killed *S. aureus*, respectively. Simultaneous administration of anti-TNF-α monoclonal antibody (mAb) and IL-1 receptor antagonist (IL-1ra) with *S. aureus *resulted in significant inhibition (80%) of 12-hour leukocyte infiltration. However, leukocyte infiltration at 24 hours and beyond and the loss of proteoglycan in *S. aureus-*induced arthritis were not affected by anti-TNF-α mAb, IL-1ra, or their combination. These results suggest that TNF-α and IL-1β involvement in the pathogenesis of *S. aureus*-induced arthritis may be limited to the initial phases of inflammation. The authors suggested that suppressing TNF-α and IL-1 may not be effective in the clinical treatment of Gram-positive bacteria-induced arthritis.

With respect to the molecular pathways involved in *S. aureus*-induced MMP expression in fibroblasts, our results suggest that *S. aureus *components could use a pathway(s) similar to that of IL-1β/TNF-α given that the MMP expression pattern, MAPK gene expression, and phosphotyrosine levels were similar in fibroblasts treated with *S. aureus *components or IL1β/TNF-α. It is also important to note that *S. aureus *is capable of inducing synthesis of inflammatory cytokines such as IL-1β and TNF-α from host cells [13]. Whether the MMP induction in fibroblasts by *S. aureus *component(s) is due to the cytokine/chemokine induced by *S. aureus *is not known at present. Previous studies by Wang and colleagues [31] have shown that inhibitors of p38 MAPK (SB202190) and ERK1/2 (PD98059) and inhibitors of Src Tyrosine kinase (PP2) and PI3-K (LY294002) effectively blocked PGN-mediated MMP-9 upregulation in neutrophils. The potential involvement of the Toll-like receptor (TLR)-2 in *S. aureus *PGN-induced joint inflammation and destruction was postulated in a study by Kyburz and colleagues [18]. Cultured synovial fibroblasts obtained from patients with RA or OA were stimulated with PGN. The expression of various integrins was determined by fluorescence-activated cell sorting. TLR-2 and MMP mRNAs as measured by real-time PCR were upregulated in fibroblasts treated with staphylococcal PGN. The levels of IL-6 and IL-8 in the culture supernatants were also increased by treatment with PGN. We demonstrated that cultured synovial fibroblasts express low levels of TLR-2 and TLR-9 mRNA. Anti-TLR-2 mAbs significantly inhibited production of IL-6 and IL-8 induced by stimulation with PGN. The authors concluded that bacterial PGNs activate synovial fibroblasts, partially via TLR-2, to express integrins, MMPs, and proinflammatory cytokines. There was no mention regarding MMP expression after TLR blockade, and it remains unclear whether TLR is involved in MMP expression in a more direct way. Our preliminary results have shown that *S. aureus *culture supernatant and whole cell lysate induce the mRNA expression of several members of the TLR family, including TLR-2 (data not shown).

To elucidate the MMP induction by *S. aureus*, we turned to two well-characterized mutant strains of *S. aureus *lacking *Sar A *and *Agr*. *Agr *and *Sar *are the two best-characterized loci responsible for modulating the expression of *S. aureus *virulence factors [38]. *S. aureus *strains lacking either locus have been shown to result in attenuation of *S. aureus *in several models of staphylococcal diseases [39-41]. Recent investigations by Blevins and colleagues [19] have also shown that mutation of S*ar A *and/or *Agr *caused reduced capacity to induce both SA and osteomyelitis. The exact mechanisms of reduced effectiveness of *Sar/Agr *mutants to cause SA or osteomyelitis are not known. Studies by Nilsson and colleagues [41] showed that mice inoculated with the *Sar A*^+ ^staphylococcal strain exhibited a more pronounced T- and B-lymphocyte activation and higher levels of serum IL-6 and IFN-γ, compared with a *Sar A*^-/- ^mutant, and infection with *Sar A*^+ ^staphylococci induced pronounced weight loss as well. These studies suggested that S*ar A *locus might control molecules that are important virulence determinants in the induction and progression of SA. We therefore tested the MMP-1, -3, and -13 expression patterns in response to *Sar*, *Agr*, or *Sar/Agr *mutants in human dermal fibroblasts. The three MMPs were selected due to their known involvement in various models of arthritis and their respective degrading actions on collagen type I, II, and III and proteoglycan, which are important constituents of connective tissues and cartilage of the joints. Our results did not show any significant differences in MMP-1 and MMP-3 mRNA levels, and 13 mRNA levels were minimal and could not be quantified with reasonable accuracy in dermal fibroblasts upon exposure to culture supernatants or cell lysates obtained from the mutants and isogenic parent strain (Figure 6). However, interestingly, the expression of TIMPs was notably increased in fibroblasts treated with *Sar/Agr *mutants compared with isogenic parent strain (Figure 7a,b). This could mean that the effective biologically active MMPs are less abundant in cells treated with the *Sar/Agr *mutants compared with cells treated with isogenic parent strain. It will be necessary to estimate the levels of biologically active MMPs (by zymograms) to determine the net effect of *Sar/Agr *mutants on MMP expression. Temporal estimation of biologically active MMPs in the joints after infection with isogenic parent and mutants will help to clarify the issue of MMPs as a factor in the observed differences in severity of diseases caused by wild-type and mutant strains. Bacteria may secrete proteolytic enzymes such as the thermolysin family secreted by *Pseudomonas aeruginosa *and *Vibrio cholera *which activate pro-MMP-1, -8, and -9 [42]. Also, proteases from the oral pathogen *Porphyromonas gingivalis *activate MMP-1, -3, and -9 [43]. If the bacterial-derived proteases are required for virulence, such proteases can be attractive therapeutic targets because their inhibition can be achieved without affecting the normal expression and function of MMPs. There are reports of other staphylococcal virulence factors associated with the pathogenesis and severity of SA [43-45]. Whether these virulence factors are associated with MMP/TIMP expression remains to be seen.

In addition to the bone and joint infections, *S. aureus *is also the prime causative agent in many skin and soft tissue infections (SSTIs), which can be manifested as superficial to deep-seated and at times become life-threatening [46-48]. Due to lack of validated clinical evidence, it is often difficult to recommend general treatment options [49]. The pathogenesis of SSTI is not understood well, and the treatment is guided mostly by epidemiological pattern and microbiological information [50]. Due to the emergence of MRSA, it is important to understand the mechanisms of tissue destruction in soft tissue infections which could lead on the identification of novel therapeutic targets. Our current *in vitro *data and the *in vivo *data reported previously by others implicate that host-derived metalloproteinases could be involved, at least in part, in tissue destruction. Excessive expression of these metalloproteinases induced by *S. aureus *could lead to the destruction of the soft tissue connective tissue architecture.

## Conclusion

We have shown that *S. aureus *is a potent inducer of multiple MMPs in human dermal and synovial fibroblasts. Our studies also indicate that MAPK-mediated signal transduction pathway involving proteins that are phosphorylated at tyrosine residues might play a role in *S. aureus*-induced MMP expression. Enhanced expression of immunoreactive MMPs by cell lysate obtained from *S. aureus *grown in the presence of rhIL-1 indicates that an inflamed milieu such as RA synovium might augment the MMP induction potential of *S. aureus*. More specific identification of the component(s) of *S. aureus *involved in the upregulation of MMP and associated signal transduction pathways may help in identifying novel targets for intervention. Based on our results, we propose that biologically active MMPs (reflected as a ratio of MMPs to TIMPs) induced by *S. aureus *could potentially accelerate the joint destruction in SA.

## Abbreviations

Agr = accessory gene regulator; AHO = acute hematogenous osteomyelitis; AMV = avian myeloblastoid virus; AP-1 = activation protein; ATCC = American Type Culture Collection; DMEM = Dulbecco's modified Eagle's medium; ELISA = enzyme-linked immunosorbent assay; ERK = extracellular signal regulated kinase; FBS = fetal bovine serum; GAPDH = glyceraldehyde phosphate dehydrogenase; IFN = interferon; IL = interleukin; IL-1ra = interleukin-1 receptor antagonist; JNK = c-jun N-terminal kinase; LT = lymphotoxin; mAb = monoclonal antibody; MAPK = mitogen-activated protein kinase; MMP = matrix metalloproteinase; MRSA = multidrug-resistant *Staphylococcus aureus*; OA = osteoarthritis; PCR = polymerase chain reaction; PGN = peptidoglycan; RA = rheumatoid arthritis; RT-PCR = reverse transcription-polymerase chain reaction; SA = septic arthritis; Sar = staphylococcal accessory regulator; SSTI = skin and soft tissue infection; TIMP = tissue inhibitor of metalloproteinases; TLR = Toll-like receptor; TNF = tumor necrosis factor.

## Competing interests

The authors wish to state that they have no commercial or other association (such as pharmaceutical stock ownership, consultancy, advisory board membership, patents, or research funding) that might contribute to competing interests.

## Authors' contributions

SK conceived the idea, designed the study, and prepared the manuscript draft. AP, AK, KH, and DS all helped in the experimental design and data analysis and provided creative and critical suggestions after reviewing the manuscript. MS provided insight into the murine SA model, provided the mutant and isogenic strains of *S. aureus*, and critically reviewed the manuscript. WA assisted with the RT-PCR work, cell culture maintenance, MMP protein arrays, as well as in review of literature. All authors read and approved the final manuscript.
